# A novel technique of rotator cuff repair using spinal needle and suture loop

**DOI:** 10.1186/1758-2555-2-28

**Published:** 2010-11-09

**Authors:** Nasir Muzaffar, Jung-Ro Yoon, Youngbae B Kim

**Affiliations:** 1 Hospital for Bone and Joint Surgery, Barzalla, Srinagar, Kashmir, India; 2 Seoul Veterans Hospital, Seoul, South Korea

## Abstract

**Background:**

We present a simple technique of arthroscopic rotator cuff repair using a spinal needle and suture loop.

**Methods:**

With the arthroscope laterally, a spinal needle looped with PDS is inserted percutaneously into the shoulder posteriorly and penetrated through the healthy posterior cuff tear margin. Anteriorly, another spinal needle loaded with PDS is inserted percutaneously to engage the healthy tissue at the anterior tear margin. The suture in the anterior needle is then delivered into the suture loop of the posterior needle using a suture retriever. The posterior needle and loop are then pulled out carrying the anterior suture with it. The two limbs of this suture are then retrieved through a cannula for knotting. The same procedure is then repeated for additional suturing. Suture anchors placed over the greater tuberosity are used to complete the repair.

**Conclusion:**

This is an easy method of rotator cuff repair using simple instruments and lesser time, hence can be employed at centers with less equipment and at reduced cost to the patient.

## Background

With rapid advances in the field of sports medicine, many pathologies hitherto inaccessible to arthroscopic surgery have now been rendered to the realm of the arthroscopist. Rotator cuff tears have been seen in this purview ever since Wolf [1-4] did the first all arthroscopic repair. Many techniques have been enumerated [3,5,6,8,9] and they require a long learning curve and numerous expensive instruments. The objective in all methods is to have a stable suture-cuff and suture-bone interface that permits early and adequate rehabilitation which would hasten the recovery and return to work and sport. The commonly used methods in the U- or L-shaped tears employ either the 2-step or the 1-step techniques [1]. In the former, the sutures are passed through each cuff margin one at a time and in the latter, through both margins simultaneously. The first method requires specialized instrumentation and takes more time while the second may result in incomplete repair since it requires the margins to be properly aligned prior to repair. The need is to have a technique combining the advantages of both that would be technically easier, use less instruments and time and give good fixation. We have tried to address these issues in our technique. The advantages that our technique offers are:

1. It is a simple, cost and time saving percutaneous technique using spinal needles employing the best possible site of insertion for a particular morphology of tear without using additional portals which may add to the operating time and morbidity.

2. The needle can be inserted perpendicular to the tear margin which gives the best possible strength of the stitch to withstand tear-out and maintains reduction of the repair.

3. Only commonly used spinal needles and suture material is employed in repair.

## Method

**Figure 1:**
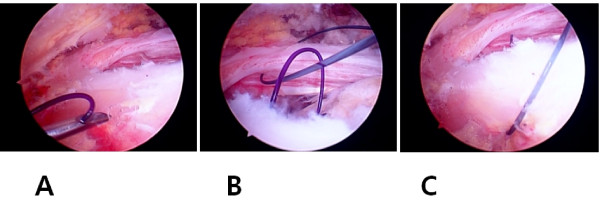
**Figure showing tendon to tendon repair with (A) the penetration of the posterior cuff margin by the spinal needle with the suture loop; (B) retrieval of the PDS suture from the anterior cuff margin using the same loop, and (C) passage of the PDS through the entire rotator cuff**.

**Figure 2:**
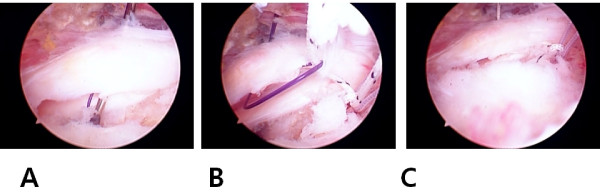
**Figure showing tendon to bone repair with (A) the penetration of the anterior cuff margin by the spinal needle with the suture loop; (B) retrieval of the suture anchor thread using the suture loop anteriorly, and(C) completed passage of all the suture anchor threads through the rotator cuff tear**.

**Figure 3:**
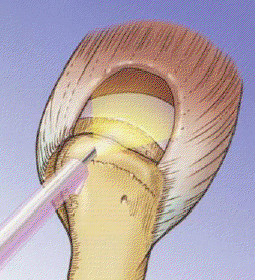
**Diagnostic arthroscopy confirming the U shaped cuff tear**.

**Figure 4:**
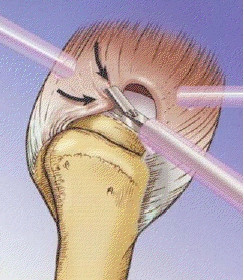
**With portals made and cannulae in place, traction is applied with a grasper to the edge of the tear to achieve concentric reduction**.

**Figure 5:**
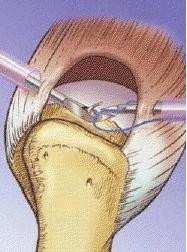
**Penetration of the loop of PDS through the posterior cuff margin**.

**Figure 6:**
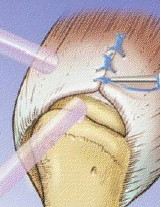
**Completion of the sutures with the SMC knot**.

**Figure 7:**
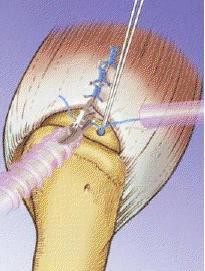
**Application of suture anchors to the greater tuberosity**.

**Figure 8:**
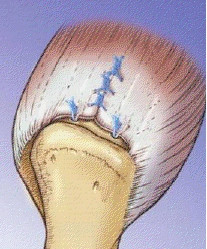
**Completed repair of the rotator cuff tear**.

We perform the procedure (Figure 1, 2) under general anesthesia in the lateral decubitus position. The lateral position is preferred because it provides a stable positioning of the patient and a pronounced glenohumeral and subacromial opening through traction on the upper limb. First, routine diagnostic arthroscopy using the standard anterior and posterior portals (Figure 3) is performed to examine the glenohumeral joint and confirm the presence of a rotator cuff tear. We also employ the lateral portal as needed. The tear margins are debrided with a shaver and any other joint pathology is noted and addressed to, if possible. Once a tear is identified and considered appropriate for repair by this method, the arthroscope is introduced via the lateral portal and subacromial decompression and acromioplasty are done. Cannulas are placed in the anterior and posterior portals for using instruments, suturing and minimizing extravasation of irrigation fluid to the surrounding soft tissues (Figure 4). A spinal needle loaded with a No 0 PDS folded on itself to form a loop is percutaneously inserted into the subacromial area posteriorly and under arthroscopic guidance, the tip of the spinal needle with the suture loop is guided to the posterior cuff margin and penetrated across it near the apex of the tear (Figure 5). Then another spinal needle loaded with No 0 PDS till the tip is inserted either percutaneously or through the anterior portal, whichever is more convenient depending upon the tear morphology and the healthy tissue of the anterior margin is penetrated and the suture advanced through the needle lumen. Next, this suture is maneuvered through the loop of suture from the posterior needle and by gently pulling on this loop and needle, the suture is extricated posteriorly. Now we have a PDS suture that has gone through the full thickness of the torn cuff and out of the joint. Then, using a suture retriever, the two limbs of the suture are retrieved through one of the two cannulas and a SMC knot (Figure 6) applied and tied down to the cuff to achieve margin convergence. This procedure is then followed by applying additional sutures to the tear in a similar fashion. In cases where the torn edge of the cuff located far medially under the acromion, the tear edge location may prevent accurate passage of the needle. We either pass a single loop through the tear edge separately and use the free ends for traction till adequate reduction is achieved or do not employ the spinal needle technique if it is too difficult. Subsequently, the region of the greater tuberosity of the humerus is abraded with a full-radius shaver/burr to create a bed of bleeding bone to promote healing of the cuff to the tuberosity, one or two suture anchors are placed on the decorticated greater tuberosity and their sutures are also retrieved utilizing the suture loop and needle (Figure 7). The sutures are then tied preferably with one suture proximal to the horizontal suture placed earlier which then acts as a check rein for the suture anchor. Thus the cuff is compressed onto the tuberosity as well as having multiple side-to-side sutures in the tear (Figure 8).

## Discussion

Arthroscopic rotator cuff repair is gaining favor with surgeons due to lower morbidity to the surrounding soft-tissue envelope, no deltoid detachment, better visualization of the pathology, better rehabilitation and improved results. However, repairs are technically demanding and need adequate visualization with additional portals (like the rear viewing portal in massive cuff tears) and special instruments to get correct orientation of the sutures which usually take several steps[3,5,6,8,9]. Millet [10] et al advocated a double anchor footprint with a mattress suture technique. Castagna [5] reported a method using a triple loaded suture anchor in the Alex stitch. Burkhart et al proposed placement of multiple simple sutures for convergence to distribute the load evenly over multiple fixation points and thus reduce the chances of stress failure and tear propagation [3]. Burkhart termed "margin convergence" to describe the observation that during side-to-side repair the surgeon can visualize the free margin of the tear converging toward the greater tuberosity with each suture being placed and that using margin convergence in the repair of U-shaped tears decreases the amount of strain at the tendon bone interface of the repair and therefore should be protective to the tendon bone interface of the repair [1]. He described a classification for rotator cuff tears as being either crescent-shaped or U-shaped tears [4]. According to Burkhart, the crescent-shaped tear is a disruption of the tendinous insertion from the greater tuberosity of the humerus without a large element of retraction. The U-shaped tear usually appears on initial inspection to be a large retracted tear often medial to the level of the glenoid. Surgical treatment of full-thickness rotator cuff defects has primarily focused on recreating the anatomy of the intact rotator cuff with reinsertion and fixation of the tendon to the greater tuberosity of the humerus using different types of instrumentation [11]. This is to be done with minimal soft tissue trauma so that the envelope integrity is maintained as much as possible. The creation of flaps or "dog ears" indicates a noncongruent repair and tension mismatch due to too much squeeze on the cuff margins that will probably fail under cyclic loading [12]. The strength of the fixation of the tendon to its insertion is of paramount importance for eventual success of the repair hence the need for proper fixation of the suture anchors [10]. We employ the routine anterior, posterior and lateral portals for the procedure and use the needles as per the convenience and expertise of the surgeon, obviating the need for additional portals or using more instrumentation via these portals which may cause additional trauma without getting concentric reduction. Also, revision of the suturing if needed (as in the case of "dog earing", which should not be accepted) is easy as the technique can be repeated till the surgeon is completely satisfied with his work since the tissue trauma is minimal. However, this technique has pitfalls and is not a panacea for all tears and we believe that tears which do not allow for adequate manipulation of the needles, for example, far medial and subacromial should preferably not be repaired with needles as suture placement will not be accurate. There is also a possibility of suture breakage when the needle with PDS loop is passed through the hard tendon cuff. This is more liable if repeated attempts are made so if repetitious attempts are employed, the suture loop should be changed. Any broken suture should be promptly retrieved with a grasper.

## Conclusions

Our all-arthroscopic technique employs simple spinal needles instead of specialized instrumentation for closure of the tears. Also, the numerous steps involved in the repair are lessened so there is a reduction in operating time. All this translates into cost benefits for the hospital and the patient. The needle being under adequate control of the operating surgeon, the cuff margins can be penetrated at a right angle which gives better grip on the tissue and as the needle can be introduced percutaneously, the need for additional portals is obviated and any suture material can be used. Also, with the passage of each suture, the result of the suture on the entire cuff can be arthroscopically assessed. We use the SMC knot [7] which has excellent knot-holding characteristics with least chances of slipping. This technique combines the advantages of side-to-side suturing of the cuff margins with the tendon-to-bone fixation in lesser time and at a lower cost. The technique is simple, easy to understand and replicate.

## Competing interests

Each author certifies that he has no commercial associations (e.g. consultancies, stock ownership, equity interests, patent/licensing arrangements, etc.) that might pose a conflict of interest in connection with the submitted article.

## Authors' contributions

All the authors contributed towards the clinical and surgical endeavour and in drafting of the manuscript and have given final approval of the version to be published.
